# Functional evidence for a de novo mutation in *WDR45* leading to BPAN in a Chinese girl

**DOI:** 10.1002/mgg3.858

**Published:** 2019-07-22

**Authors:** Qiuhong Xiong, Wenjing Li, Ping Li, Zhonghua Zhao, Changxin Wu, Han Xiao

**Affiliations:** ^1^ Institutes of Biomedical Science Shanxi University Taiyuan China

**Keywords:** autophagy, BPAN, novel mutation, WDR45

## Abstract

**Background:**

Beta‐propeller protein‐associated neurodegeneration (BPAN, OMIM 300894) is an X‐linked neurodegenerative disorder caused by mutations in *WDR45*. WDR45 is required for autophagy, defect in WDR45 impaired autophagy which contributes for the pathogenesis of BPAN. Previously, we reported a novel de novo mutation (c.1040_1041del, p.Glu347GlyfsTer7) in *WDR45* (NM_007075) in a 3‐year‐old Chinese girl with BPAN.

**Methods:**

The protein structure was constructed using SWISS‐MODEL and the isoelectric point (pI) was predicted by the online pI/Mw tool at ExPASy. The functional effects of this mutation were predicted by two online software programs: PROVEN and MutationTaster. Stable overexpression of Flag‐tagged wild‐type or mutant *WDR45* in HeLa cells was constructed. Protein levels of LC3 and p62 were analyzed by western blot upon treatment with/without autophagy inhibitor Bafilomycin A1, the formation of LC3 puncta were analyzed in HeLa cells transfected with mCherry‐LC3 by confocal microscopy.

**Results:**

The mutation resulted in a shift of pI from 6.74 to 8.84 and was predicted to be pathogenic. The protein levels of LC3‐II and p62 were increased in cells overexpression of wild‐type and mutant *WDR45* while the protein levels were not increased in cells overexpression of mutant *WDR45* upon treatment with autophagy inhibitor Bafilomycin A1. Results from confocal microscopy revealed that LC3‐positive puncta were increased in cells expressing both wild‐type and mutant *WDR45* while the number of LC3‐positive puncta was not increased in cells expressing mutant *WDR45* upon treatment with Bafilomycin A1.

**Conclusion:**

Our study evidenced that this novel mutation in *WDR45* impaired autophagy in cells thus this mutation is the cause for BPAN in this patient.

## 1 INTRODUCTION

Beta‐propeller protein‐associated neurodegeneration (BPAN, OMIM 300894), a subtype of neurodegeneration with brain iron accumulation (NBIA), is an X‐linked neurodegenerative disorder caused by mutations in *WDR45*, the gene encoding a member of WIPI family, WDR45 (also called WIPI4). Affected individuals are characterized by global developmental delay in early childhood that is essentially static, with slow motor and cognitive gains until adolescence or early adulthood. In young adulthood, affected individuals develop progressive dystonia, parkinsonism, extrapyramidal signs, and dementia resulting in severe disability. Brain MRI of BPAN patients also show iron accumulation in the globus pallidus and substantia nigra (Haack et al., 2012). Recently, our group reported a 3‐year‐old female patient with febrile seizures, cognitive and motor developmental delay, iron deposition in globus pallidus, and thin corpus callosum. It was also confirmed that the patient have a novel de novo mutation in the *WDR45* gene (NM_007075, c.1040_1041del, p.Glu347GlyfsTer7) which was not found in her parents and sister (Xiao et al., 2018). This study confirmed that this mutation in this patient is pathogenic and impairs autophagy, we conclude that this mutation in *WDR45* is the cause for BPAN in this patient.

## 2 MATERIALS AND METHODS

### 2.1 Sequence analysis

The *WDR45* mutation was found by whole‐exome sequencing and validated by PCR and then Sanger sequencing by our group as described (Xiao et al., 2018). *WDR45* c.1040_1041del mutation caused amino acid changes at Glu347, and resulting in frameshift and premature truncation of the protein. The structures of WDR45 were constructed by SWISS‐MODEL (http://www.swissmodel.expasy.org) using 6iyy.1 as the protein structure template (Liang, Ren, Zhang, & Feng, 2019). The isoelectric point (pI) of wild‐type and mutant WDR45 was predicted by the online pI/Mw tool at ExPASy, (https://web.expasy.org/compute_pi/). The functional effects of this mutation were predicted by two online software programs: PROVEN (http://provean.jcvi.org/index.php; Choi, Sims, Murphy, Miller, & Chan, 2012) and MutationTaster (http://www.mutationtaster.org/; Schwarz, Cooper, Schuelke, & Seelow, 2014).

### 2.2 Vector construction

The full‐length open reading frame of human *WDR45* (NM_007075) was PCR‐amplified from cDNAs obtained from HEK293 cells using primers: 5'‐CGCCTCGAGATGACTCAACAGCCACTTCGAG‐3' and 5'‐CGCGAATTCCTTAAAAGTCATCATCATCACAG‐3'; mutant *WDR45* was PCR‐amplified using primers: 5'‐CGCCTCGAGATGACTCAACAGCCACTTCGAG‐3' and 5'‐CGCGAATTCTCAAGGTACACGTCGAAAGCCTCTGTTGCAGTTTCCATC‐3'; human LC3 (NM_022818.5) was PCR‐amplified using primers: 5'‐CGCGAATTCATGCCGTCGGAGAAGACCTT‐3' and 5'‐CGCCTCGAGTTACACTGACAATTTCATCCCGAAC‐3';

### 2.3 Cell culture and cell transduction

HeLa cells were cultured at 37°C and 5% CO_2_ in Dulbecco's modified Eagle medium (Life Technologies) supplemented with 10% fetal bovine serum (Gibco) and 1% penicillin/streptomycin (Solarbio Life Sciences). The stably expressing Flag, wild‐type and mutant Flag‐*WDR45* cells were constructed by lentiviral transduction. Forty‐eight hours postinfection, the culture medium was supplemented with 2 μg/ml puromycin (Solarbio Life Sciences), cells were selected for 2 weeks and confirmed by western blot. Transient transfection of cells with mCherry‐LC3 was conducted using Lipofectamine 2000 (Invitrogen) according to the manufacturer's instructions.

### 2.4 Western blot analysis

Cells were incubated in DMEM medium in the presence or absence of 300‐nM Bafilomycin A1 (Solarbio Life Sciences) for 3 hr, then cell pellets were extracted using RIPA lysis buffer (10‐mM Tris pH7.4, 150‐mM NaCl, 1% NP‐40, 0.5% sodium deoxycholate, 1‐mM PMSF, and protease inhibitor cocktail). Gels were blotted onto PVDF membranes. The primary antibodies used for western blotting were anti‐Actin (1:10000; proteintech, China), anti‐LC3 (1:3000; Abcam), anti‐p62 (1:2000; Abcam), and anti‐Flag M2 (1:1000; Sigma), the secondary antibodies used for western blotting were anti‐rabbit IgG, horseradish peroxidase (HRP)‐linked (1:10000; proteintech) or anti‐rabbit IgG, CF680 (1:10000; Sigma), anti‐mouse IgG, HRP‐linked (1:10000; proteintech) or anti‐mouse, Alexa Fluor 790 (1:10000; Abcam).

### 2.5 Fluorescence microscopy

Cells were grown on sterile coverslips and fixed in 4% formaldehyde for 15 min at room temperature and blocked with PTB butter (PBS containing 0.1% Triton X‐100 and 0.1% BSA). Nuclei were stained with 4',6‐diamidino‐2‐phenylindole (DAPI, Sigma). Images of fixed cells were taken with a Zeiss LSM710 Microscope with a 63 X 1.4 DIC Plan‐Apochromat oil‐immersion objective.

### 2.6 Statistical analysis

The number of punctate structures per cell was quantified using ImageJ, western blot analysis was performed at least three times and data were presented as mean + *SE*, statistical analysis was done by *t* test, the significance level for all tests was considered *p* < .05.

## 3 RESULTS AND DISCUSSION

**Figure 1:**
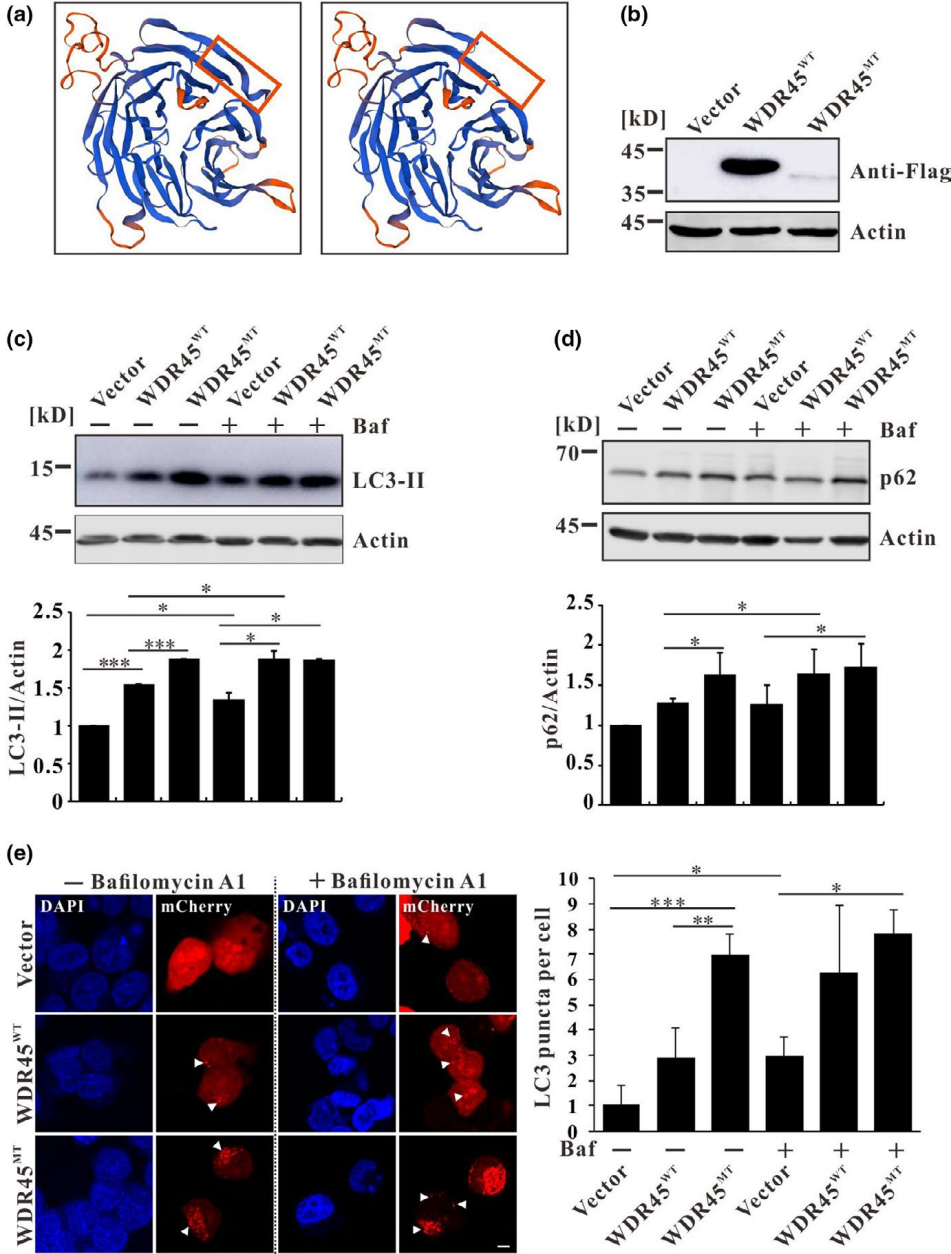
(a) SWISS‐MODEL predicted structures of wild‐type and mutant WDR45 based on the structure of WIPI3. This mutation resulted in a truncated WDR45 which was indicated by the red box. (b) Flag‐tagged protein was confirmed by western blot in stable cell lines expressing wild‐type or mutant *WDR45*. (c) LC3 protein level was detected by western blot in stable expressing Flag, Flag‐WDR45^WT^, or Flag‐WDR45^MT^ HeLa cell lines, LC3‐II signal was quantified by densitometric analysis and normalized based on the signal of Actin. Bars represent mean values and standard errors of three independent experiments. The asterisk represents significant difference determined by Student's *t* test. *p < .05, **p < .01, ***p < .001. (d) p62 protein level was detected by western blot. p62 signal was quantified by densitometric analysis and normalized based on the signal of Actin. Bars represent mean values and standard errors of five independent experiments. The asterisk represents significant differences determined by Student's *t* test. *p < .05, **p < .01, ***p < .001. Baf: Bafilomycin A1. (e) Fluorescence analysis of stable HeLa cell lines transfected with mCherry‐LC3 and mean numbers of mCherry‐LC3 puncta per cell were calculated by ImageJ (least 15 cells, *n* = 3). The asterisk represents significant difference determined by Student's *t* test. *p < .05, **p < .01, ***p < .001. Baf: Bafilomycin A1. Scale Bar: 2 μm

The C‐terminal of WDR45 is highly conversed among various species (Xiao et al., 2018). The deletion of two base pairs leading to a frameshift, which resulted in a truncated protein (Figure 1a), and the pI of the protein convert form 6.74 to 8.84 which was predicted by the online pI/Mw tool at ExPASy (https://web.expasy.org/compute_pi/). The charge of wildtype protein is negative under normal physiological condition, while the charge of mutant protein will change to positive which should affect the interactions of WDR45 with its partners. Using program PROVEN to predict to be damaging with a score of −48.651, where scores equal to or below −2.5 are considered deleterious. MutationTaster also predicted that the alteration was disease‐causing. In addition, no other mutations were observed in other NBIA‐related genes (Xiao et al., 2018). Therefore, we suggest that this mutation (c.1040_1041del, p.Glu347GlyfsTer7) in *WDR45* is very likely to cause BPAN in this patient.

WDR45 is an ortholog of yeast ATG18 which is essential for autophagy. Saitsu et al. (2013) identified five different de novo heterozygous truncating mutations in *WDR45* gene in five unrelated female patients with BPAN. All the patient cells showed impaired autophagy suggested that impairment of autophagy contributes for the pathogenesis of BPAN (Saitsu et al., 2013). To test if this mutation is pathogenic, HeLa cells stably expressing Flag, Flag‐WDR45^WT^, or Flag‐WDR45^MUT^ were constructed (Figure 1b). Western blotting analyses showed a higher amount of lipidated LC3 (LC3‐II) in the cells transfected with both wild‐type and mutant *WDR45* compared with the cells transfected with empty vector, and the protein level of LC3‐II in WDR45^MT^‐expressing cells was even higher than that in WDR45^WT^‐expressing cells (Figure 1c). As WDR45 promotes autophagosome formation (Bakula et al., 2017), overexpression of WDR45^WT^ would promote the lipidation of LC3. The increased protein level of LC3‐II in WDR45^MT^‐expressing cells suggests an inhibition of autophagy. To confirm this, cells were treated with autophagy inhibitor Bafilomycin A1 which blocks autophagosome fusion with lysosome. Western blot revealed that LC3‐II was accumulated in WDR45^WT^‐expressing cells but not in WDR45^MT^‐expressing cells upon treatment with Bafilomycin A1 (Figure 1c), suggesting that Bafilomycin A1 blocked autophagic flux thus leading to an accumulation of LC3‐II in WDR45^WT^‐expressing cells while WDR45^MT^ already inhibited autophagy; thus there is no significant increase of LC3‐II in WDR45^MT^‐expressing cells. Furthermore, the protein level of p62, which is a substrate of autophagy, was also increased in WDR45^WT^‐expressing cells but not in WDR45^MT^‐expressing cells upon treatment with Bafilomycin A1 suggesting that mutant WDR45 inhibited autophagy (Figure 1d). Consistent with the western blot analysis, images taken by confocal microscopy demonstrated the increment of LC3‐containing puncta in the HeLa cells expressing wild‐type and mutant *WDR45* compared with that observed in the cells expressing empty vector (Figure 1e). Upon treatment with Bafilomycin A1, the LC3‐positive puncta were increased in WDR45^WT^‐expressing cells but not increased in WDR45^MT^‐expressing cells (Figure 1e). Taken together, all these results suggest that this mutation in *WDR45* (c.1040_1041del, p.Glu347GlyfsTer7) impairs autophagy in cells.

Our data show that a de novo mutation in *WDR45* (c.1040_1041del, p.Glu347GlyfsTer7) leads to a truncated protein and a change of the protein pI, experimental results revealed that this mutation in *WDR45* impaired autophagy in cells. In conclusion, our results suggest that this mutation in *WDR45* leads to autophagy deficiency as a cause for BPAN in this patient.

## CONFLICT OF INTEREST

None declared.
